# Sex modifies the renal consequences of high fructose consumption introduced after weaning

**DOI:** 10.3389/fphys.2023.1090090

**Published:** 2023-03-15

**Authors:** Letícia Maria Monteiro, Celine Farias Barbosa, Débora Conte Kimura Lichtenecker, Rogério Argeri, Guiomar Nascimento Gomes

**Affiliations:** ^1^ Department of Physiology, Escola Paulista de Medicina, Federal University of São Paulo, São Paulo, Brazil; ^2^ Postgraduate Program in Translational Medicine, Department of Medicine, Escola Paulista de Medicina, Federal University of São Paulo, São Paulo, Brazil

**Keywords:** renal function, fructose overload, sexual dimorphism, electrolyte excretion, blood pressure

## Abstract

After lactation, many children consume fructose-rich processed foods. However, overconsumption of these foods can predispose individuals to non-communicable chronic diseases, which can have different repercussions depending on the sex. Thus, we evaluated the effects of fructose overload introduced after weaning on the renal function of young rats of both sexes.

**Methods**: After weaning, male and female offspring of Wistar rats were assigned to drink water (the male/water and female/water groups) or 20% D-fructose solution (male/fructose and female/fructose groups). Food and water or fructose solution was offered *ad libitum.* Rats were evaluated at 4 months. Parameters analyzed: blood pressure, body weight, triglyceride levels, glomerular filtration rate, sodium, potassium, calcium, and magnesium excretion, macrophage infiltration, and eNOS and 8OHdG expression in renal tissue. CEUA-UNIFESP: 2757270117.

**Results**: Fructose intake affected the blood pressure, body weight, and plasma triglyceride in all rats. Glomerular filtration rate was significantly reduced in males that received fructose when compared to that of the control group. Sodium and potassium excretion decreased in all fructose-treated rats; however, the excreted load of these ions was significantly higher in females than in males. In the female control group, calcium excretion was higher than that of the male control group. Fructose overload increased magnesium excretion in females, and also increased macrophage infiltration and reduced eNOS expression in both males and females.

**Conclusion**: Fructose overload introduced after weaning caused metabolic and renal changes in rats. Renal function was more affected in males; however, several significant alterations were also observed in the female-fructose group.

## Introduction

Nutrition in the early stages of child development is a determining factor of an individual’s health, with proper introduction of food after weaning being a crucial component (Hawkes et al., 2015). The consumption of inadequate food at this stage is a risk factor for the development of non-communicable diseases in the future (WHO, 2016; Porter et al., 2018). In recent decades, we have experienced significant changes in lifestyle, which has resulted in a steep increase in the consumption of ultra-processed food (UPF). Due to their formulations and presentations, UPFs tend to be consumed in excess and replace traditional, healthier foods (Blake-Lamb et al., 2016; Mameli et al., 2016; WHO, 2016; Pietrobelli et al., 2017). In addition, this new diet, consisting of UPFs, has also been offered to children less than 2 years of age in several countries (Cirino et al., 2014; Pries et al., 2016; Cediel et al., 2018; Cornwell et al., 2018). UPF contains dyes, emulsifiers, and additives such as high-fructose corn syrup (HFCS), a product with a large percentage of fructose (Martínez Steele et al., 2016). Studies have demonstrated that increased fructose consumption, which has steadily risen in recent decades, is associated with glucose intolerance and cardiovascular and renal changes (Cunha et al., 2007; Brito et al., 2008; Abdulla et al., 2011; Cabral et al., 2014; Bernardes et al., 2018). Thus, it is important to investigate the effects of this sugar intake when introduced at an early age.

Recent studies have shown that sexual dimorphism is related to the morphology and function of renal tubules (Veiras et al., 2017; Kalucki et al., 2020). Male rats appear to have a higher proportion of proximal tubules, which under the action of androgenic hormones, could lead to sodium and volume retention (Quigley, 2008). Contrastingly, the female sex has a more significant proportion of distal nephron segments, which could provide better regulation of saline excretion (Veiras et al., 2020). In addition, recent studies have shown differences in the development and progression of chronic kidney disease, depending on gender (Carrero et al., 2018; Ricardo et al., 2019).

Considering the lack of data on the long-term effects of fructose overload on renal alterations in both sexes, especially when this sugar intake is started soon after weaning, the objective of the present study was to evaluate the effect of excess-fructose diets introduced after weaning on the morphology and renal function of male and female rats in adulthood. Blood pressure was measured at the end of the experimental period.

## Materials and methods

This study was approved by the Ethical Research Committee of the Universidade Federal de Sao Paulo (protocol 2757270117) and adhered to the international guidelines for the care of animals. Wistar rats were obtained from the “Centro de Desenvolvimento de Modelos Experimentais Para Biologia e Medicina (CEDEME)” Animal Breeding Center at our university. Female rats were divided into pairs and caged overnight with a male mate. Vaginal smears were collected the following morning, and the presence of sperm was considered a positive result. After birth, litter size was standardized to eight pups per litter and the offspring remained with the dams for 21 days. After weaning, pups were separated and randomly divided into the following experimental groups:

MW, male rats that received water and food *ad libitum*;

MF, male rats that received fructose solution (20%) and food *ad libitum*;

FW, female rats that received water and food *ad libitum*;

FF, female rats that received fructose solution (20%) and food *ad libitum*.

During the experimental period, the fructose groups received a drinking solution containing 20% d-fructose (D-Fructose, Labsynth, Diadema-S.P, Brazil). The offer of fructose started at weaning (at 3 weeks of age) and continued until 4 months of age, totaling 13–14 weeks of treatment. The amount of fructose offered by different protocols varied widely (10%–60%) (Abdulla et al., 2011). The concentration used in the present study was 20% because it was relatively low while still shown to affect blood pressure (Bernardes et al., 2018).

The rats were kept in a temperature-controlled room (22°C) with lights on from 7 a.m. to 7 PM. At 4 months of age, their systolic blood pressure (BP) and weight was measured, and their renal function was evaluated. The body mass index (BMI) was calculated by the formula: BMI = body weight (g)/length^2^ (cm^2^).

### Measurement of systolic blood pressure

BP was evaluated by tail plethysmography. Rats were habituated to the procedure for 2–3 weeks before the measurements by placing them in the heated chamber (34°C) for up to 10 min and simulating the process. For measurement the sphygmomanometer with a sensor connected to a recording system (Monitor Ratpalp. b, IITC, United States) was coupled to the proximal portion of the rat tail (caudal artery). The cuff was inflated to 220 mmHg and slowly deflated, and the systolic BP was recorded. For each rat, three to five measurements were taken in a row, and the BP value was considered the average of these measurements.

### Evaluation of renal function

The rats were placed in metabolic cages for 24 h. Urine and blood samples were collected to measure creatinine, urea, and triglyceride levels, as well as sodium, potassium, chloride, calcium, and phosphate concentrations.

Plasma and urine creatinine levels were measured using the Jaffe method (Creatinine K vet, Labtest, MG, Brazil), and the glomerular filtration rate (GFR) was determined based on creatinine clearance. Plasma and urine urea levels were measured using a urea detection kit (Ureia-CE, Labtest, MG, Brazil). The concentrations of sodium, potassium, chloride, calcium, and phosphate were obtained using a Hitachi Cobas c702 analyzer (Roche Diagnostics, Indianapolis, IN, United States). The sodium and potassium fractional excretion (FE%) was calculated by the formula: FE% = (EL x 100)/FL, where EL is excreted load and FL is filtered load. The protein concentration in urine was quantified using the Bio-Rad Protein Assay (Bio-Rad Laboratories Inc, United States).

### Renal morphology

The kidney samples were fixed in Bouin’s solution (ethanol saturated with picric acid 75%, formaldehyde 20%, and acetic acid 5%) and embedded in paraffin. Five-micrometer histological sections were cut and stained with hematoxylin and eosin. Glomerular area was evaluated using a light microscope (Nikon H550L, Japan) and a camera. Images were analyzed using an image analysis software (Nikon, NISElements 3.2, Japan) and the encircled areas were determined using computerized morphometry. Twenty cortical fields were analyzed on each slide (magnification ×200). For immunohistochemical analysis, sections were incubated overnight at 4 °C with anti-CD68 for macrophage identification (anti ED1, 1:500; Serotec, Sigma-Aldrich, MO, United States), anti-endothelial nitric oxide synthase (eNOS), 1:250 (Gene-tex, CA, United States), and anti-8OHdG, 1:150 (Gene-tex, CA, United States). The reaction products were determined using a universal immuno-peroxidase polymer (Histofine-Nichirei Biosciences). For quantitative analysis, the percentage of area was assessed in 20 consecutive cortical fields for each sample (×200 magnification). Images were taken using a microscope (Eclipse 80i, Nikon, Tokyo, Japan) equipped with a digital camera (DSRi1, Nikon) and analyzed using the NIS-Elements (Nikon) software.

### Statistical analysis

Results are presented as mean ± standard error and were analyzed by two-way ANOVA. Additionally, Tukey’s *post hoc* test was used for multiple comparisons between groups (Prism 6.0, GraphPad). Values of *p* ≤ 0.05 were considered significant changes.

## Results

Fructose intake had a positive effect on blood pressure in both male and female rats (*p* = 0.0003), however the increase in female was more prominent. Fructose also influenced the body weight (*p* = 0.0134) and body mass index (*p* = 0.0039); as expected, females presented lower body weight than males. The triglycerides plasma concentration was increased under fructose consumption (*p* = 0.0004); in females, the increase in this parameter was around 100%.

Fructose consumption induced an increase in liquid (*p* = 0.0014) and caloric intake (*p* = 0.0013). These results are shown in Table 1.

**TABLE 1:** Summary of general parameters observed rats submitted to fructose overload.

Parameter	Male		Female		Two-way-ANOVA		
	Water (N = 6)	Fructose (N = 9)	Water (N = 8)	Fructose (N = 10)	Sex effect	Fructose effect	Interaction effect
**Systolic blood pressure** (mmHg)	122.5 ± 2.2	129.5 ± 2.3	116.9 ± 1.2	127.0 ± 2.1*	*p* = 0.0607	***p* = 0.0003**	*p* = 0.4687
**Body weight** (g)	366.5 ± 13.6	415.0 ± 18.1	236.2 ± 8.3^#^	255.7 ± 7.6^#^	***p* < 0.0001**	***p* = 0.0134**	*p* = 0.2694
**Kidney weight** (% of whole body)	0.81 ± 0.03	0.73 ± 0.02	0.77 ± 0.02	0.70 ± 0.02	*p* = 0.1485	***p* = 0.0025**	*p* = 0.8103
**BMI** (g/cm^2^)	0.53 ± 0.009	0.60 ± 0.023*	0.45 ± 0.012^#^	0.48 ± 0.009^#^	***p* < 0.0001**	***p* = 0.0039**	*p* = 0.2070
**Triglycerides** (mg/dL)	68.0 ± 10.7	115.0 ± 9.8	44.6 ± 5.1	95.3 ± 16.0*	*p* = 0.0894	***p* = 0.0004**	*p* = 0.8829
Daily intake							
Liquid (mL/24 h)	26.6 ± 2.1	42.7 ± 4.7*	29.0 ± 1.5	37.7 ± 3.3	*p* = 0.7050	***p* = 0.0014**	*p* = 0.3053
Food (g/24 h)	19.4 ± 1.2	12.5 ± 1.1*	18.0 ± 0.7	18.0 ± 2.1^#^	*p* = 0.1961	***p* = 0.0340**	***p* = 0.0351**
Food (g/24 h kg)	53.5 ± 4.2	30.6 ± 3.1*	76.4 ± 2.4^#^	69.0 ± 6.6^#^	***p < 0.0001***	***p = 0.0040***	*p = 0.1222*
Caloric (kcal/24 h)	53.4 ± 11.0	77.9 ± 6.6	63.1 ± 2.5	93.2 ± 8.4*	*p* = 0.1134	***p* = 0.0013**	*p* = 0.7144

Caloric intake for fructose-fed rats was calculated by adding the calories ingested as food (3.5 kcal per Gram) and as fructose solution (0.8 kcal/ml). Differences statistically significant when *p* < 0.05 (bold); vs respectively water control* or male^**#**^ using Tukey post test after two-way-ANOVA. Values are means ± standard error.

That the bold-italic values indicates the difference is statistically significant as described in the caption.

Table 2 presents the parameters of renal function. Fructose intake significantly reduced the glomerular filtration rate (creatinine clearance) (*p* = 0.0348), particularly in male rats. The blood urea concentration was equally reduced in males and females (*p* < 0.0001). Urinary volume was increased in fructose-treated groups (*p* = 0.0065). Serum concentrations of sodium, potassium, chloride, calcium, and magnesium remained normal.

**TABLE 2:** Summary of urinary and blood parameters observed in rats submitted to fructose overload.

Parameter	Male		Female		Two-way-ANOVA		
	Water (N = 6)	Fructose (N = 9)	Water (N = 8)	Fructose (N = 10)	Sex effect	Fructose effect	Interaction effect
**Glomerular filtration rate** (mL/min/kg)	8.9 ± 1.2	6.0 ± 0.7*	7.5 ± 0.4	6.6 ± 0.5	*p* = 0.5926	***p* = 0.0109**	*p* = 0.1690
**Urinary volume** (mL/24 h)	13.8 ± 0.7	25.3 ± 4.6	13.3 ± 1.2	19.6 ± 2.3	*p* = 0.3158	***p* = 0.0065**	*p* = 0.3985
**Albuminuria** (mg/24 h)	26.3 ± 2.2	30.2 ± 5.5	15.8 ± 3.5	20.9 ± 3.3	***p* = 0.0255**	*p* = 0.2847	*p* = 0.8861
Blood							
Creatinine (mg/dL)	0.28 ± 0.03	0.37 ± 0.03	0.34 ± 0.03	0.38 ± 0.03	*p* = 0.2565	***p* = 0.0348**	*p* = 0.3825
Urea (mg/dL)	45.6 ± 2.3	30.8 ± 2.6*	42.2 ± 1.7	28.3 ± 1.3*	*p* = 0.1617	***p* < 0.0001**	*p* = 0.8463
[Na^+^]_p_ (mEq/L)	142.2 ± 0.75	141.7 ± 0.73	142.0 ± 1.50	140.3 ± 0.80	*p* = 0.4554	*p* = 0.2867	*p* = 0.5584
[K^+^]_p_ (mEq/L)	4.5 ± 0.11	4.2 ± 0.13	4.6 ± 0.37	4.4 ± 0.34	*p* = 0.5234	*p* = 0.3634	*p* = 0.7670
[Cl^−^]_p_ (mEq/L)	101.0 ± 1.0	99.1 ± 1.0	102.1 ± 1.5	99.7 ± 1.0	*p* = 0.4704	*p* = 0.0771	*p* = 0.8178
[Ca^+2^]_p_ (mg/dL)	9.8 ± 0.11	10.0 ± 0.09	9.7 ± 0.14	9.9 ± 0.04	*p* = 0.2876	***p* = 0.0232**	*p* = 0.8851
[Mg^+2^]_p_ (mg/dL)	2.3 ± 0.06	2.1 ± 0.05	2.2 ± 0.06	2.1 ± 0.03	*p* = 0.6547	***p* = 0.0252**	*p* = 0.3095
[pO_4_^-^]_p_ (mg/dL)	7.3 ± 0.26	6.7 ± 0.28	6.2 ± 0.32	5.9 ± 0.23	***p* = 0.0030**	*p* = 0.1563	*p* = 0.6922

Differences statistically significant when *p* < 0.05 (bold); vs respectively water control* or male^**#**^ using Tukey post test after two-way-ANOVA. Values are means ± standard error.

**FIGURE 1:**
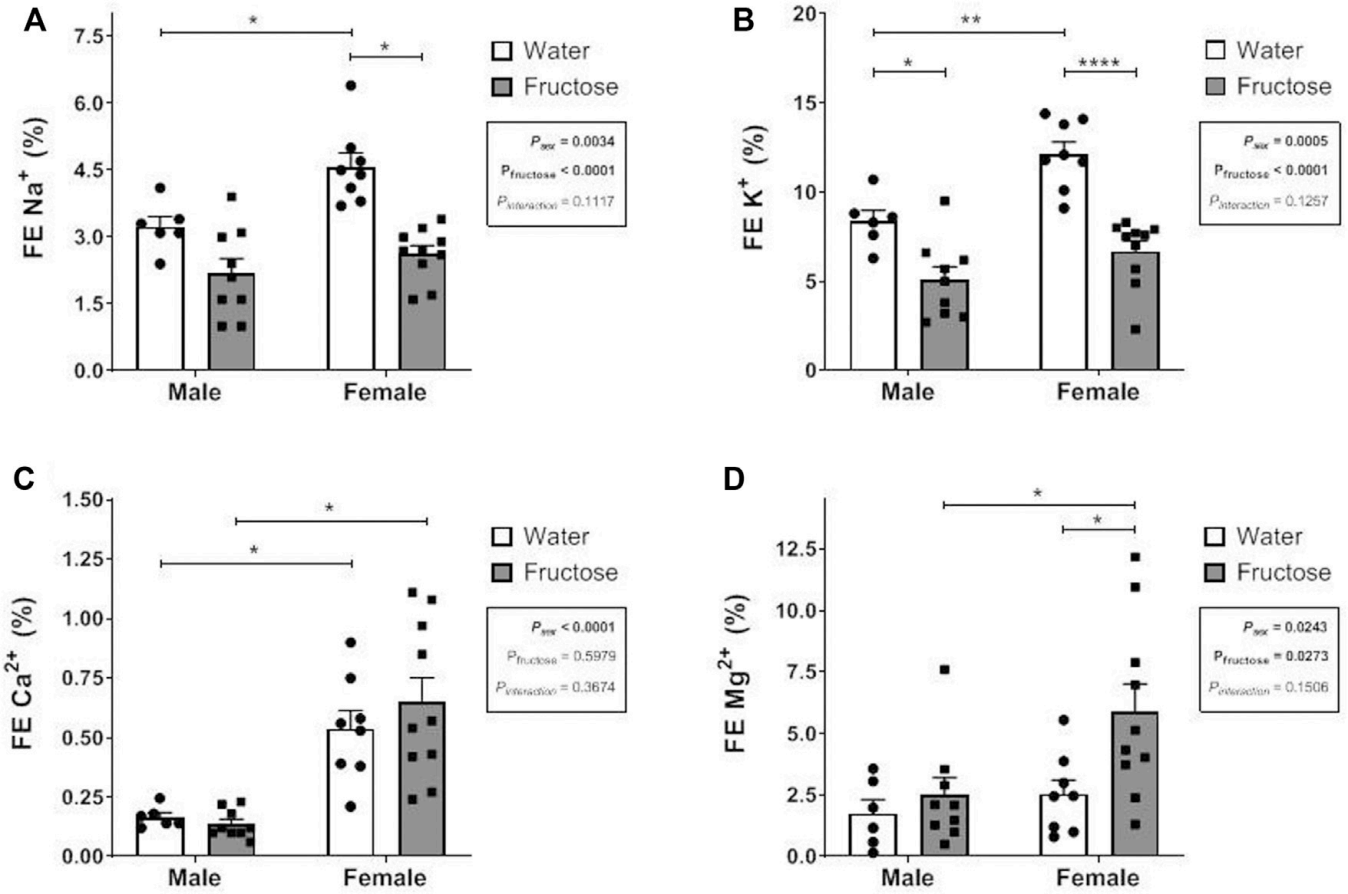
Fractional Excretion (FE) of sodium **(A)**, potassium **(B)**, calcium **(C)** and magnesium **(D)** in male and female rats that received or not fructose drinking solution. Values are mean ± standard error. Two-way ANOVA followed by Tukey’s *post hoc* test, *p ≤ 0.05; **p ≤ 0.01; ****p ≤ 0.001, 6-10 animals per group.

Figure 1 shows the fractional excretion (FE%) of the minerals in the experimental groups. Fructose intake reduced the FE% of sodium (*p* < 0.0001) and potassium (*p* < 0.0001) but increased that of magnesium (*p* = 0.0273). In the female control group, sodium, potassium, and calcium excretion levels were higher than those in the male control group. Fructose significantly increased magnesium excretion in female group.

**FIGURE 2:**
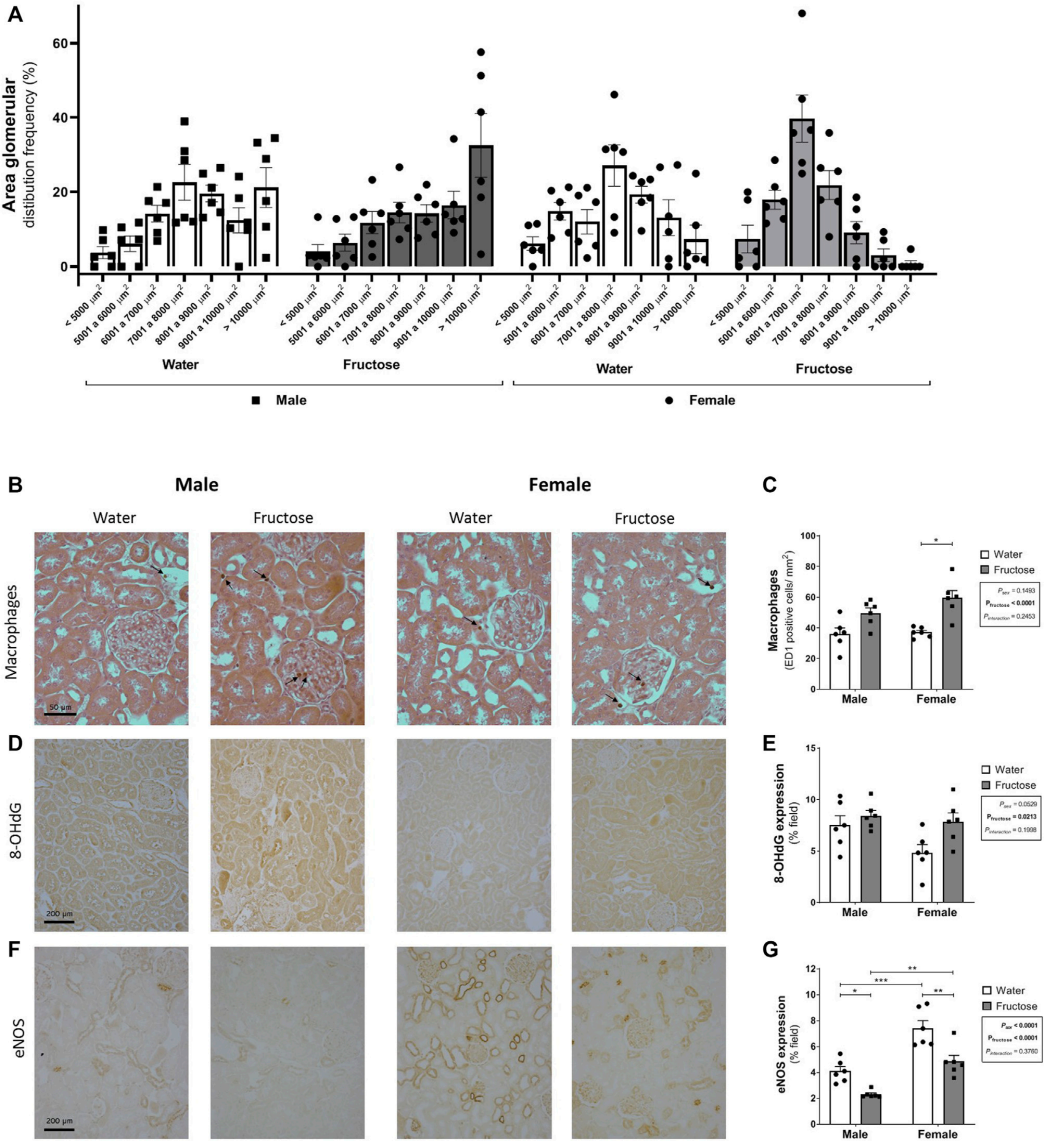
Glomerular area distribution according to the size **(A)** and expression of markers of renal dysfunction: representative photomicrographs and quantitative analysis by immunohistochemistry of the macrophages **(B–C)** (ED1 positive cells–arrows; original magnification ×400); 8-OHdG **(D–E)**; and eNOS **(F–G)** (original magnification ×200). Values are mean ± standard error. Two-way ANOVA followed by Tukey’s *post hoc* test, *p ≤ 0.05; **p ≤ 0.01; ****p ≤ 0.001, six animals per group.

Figure 2 shows the morphological parameters of the kidneys, with Figure 2A showing the glomerular area distribution according to the size (%). Female groups presented a Gaussian distribution while male groups presented a higher frequency of larger glomeruli, thus altering the profile of glomerular area distribution and resulting in an increase in the mean value of glomerular area in male group. The mean value of glomerular area for each group was: MW: 8397.6 ± 355 [*n* = 6]; MF: 8725.7 ± 384 [*n* = 6]; FW: 7599.8 ± 317 [*n* = 6]; FF: 6676.4 ± 195 [*n* = 6] µm^2^ (P_sex_ = 0.0003; P_fructose_ = 0.3695; P_interaction_ = 0.069).

Fructose intake increased macrophage infiltration (*p* < 0.0001) and the expression of 8-OHdG (*p* = 0.0213). In contrast, the expression of eNOS was downregulated by fructose (*p* < 0.0001). Female rats exhibited increased eNOS expression when compared with male rats even with fructose ingestion (Figure 2B).

## Discussion

The present study evaluated the impact of high-fructose consumption introduced after weaning on the morphology and renal function of rats of both sexes. Male rats that received fructose had a significant reduction in the glomerular filtration rate when compared to that of the male control group. In both sexes, the FE of sodium and potassium were reduced by fructose, although this excretion was significantly higher in female rats than in male rats. Fructose intake augmented magnesium excretion only in the female group. The treatment increased macrophage infiltration and downregulated eNOS expression in rats of both sexes. Additionally, fructose intake affected the body weight, body mass index, and serum triglyceride concentration, confirming the metabolic consequences of fructose overconsumption (Elliott et al., 2002; Basciano et al., 2005).

This study also confirmed the effect of excessive fructose consumption on BP. Activation of the sympathetic nervous system (SNS) has been identified as being responsible for this result (De Angelis et al., 2012; Tran et al., 2014; Soncrant et al., 2018); however, the factors that trigger SNS activation are still not well defined. High insulin levels have been suggested as a possible trigger (Klein and Kiat, 2015) as well as the direct impact of this sugar on the central nervous system, increasing local oxidative stress (Spagnuolo et al., 2020).

As fructose overload can cause autonomic imbalance, increased renal sympathetic nerve activity (RSNA) may have occurred, which in turn modifies vascular tone and renal hemodynamics, reducing GFR (DiBona, 2000, 2001; Sata et al., 2018). However, increased activation of the intrarenal renin-angiotensin system (RAS) can also lead to a reduction in GFR. This activation can occur either because of fructose overload (Yokota et al., 2018) or as a consequence of RSNA activation (DiBona, 2000, 2001; Sata et al., 2018). Additionally, increased reactive oxygen species (ROS) production may alter renal hemodynamics (Xu et al., 2020), which may be associated with the production of uric acid (Choi et al., 2010). In the plasma, uric acid is considered an antioxidant. However, inside the cell, it becomes a pro-oxidant (Sautin et al., 2007), contributing to an increase in ROS concentration by activating NADPH oxidase and reducing the activity of the eNOS enzyme and the production of nitric oxide (NO) (Ejaz et al., 2007). Further experiments are necessary to confirm the role of these mechanisms in this experimental model.

Studies indicate that a high-fructose diet can increase renal sodium reabsorption, contributing to an elevated blood pressure. The activity of NHE_3_ (Na^+^/H^+^ exchanger) in the proximal tubules of fructose-treated rats has been evaluated by Cabral et al. (2014). These authors observed that in the presence of fructose, the activity of NHE_3_ increased, and in the presence of angiotensin II, this increase was even more significant, suggesting that fructose increased the sensitivity of the proximal convoluted tubule (PCT) to angiotensin II (Ejaz et al., 2007). Xu et al. (2017) found that a high-fructose diet increases renal prorenin receptor expression, stimulating the expression of NHE_3_, NKCC2, and intrarenal RAS components. An increased expression of RAS components in the kidney tissue of fructose-treated animals was also observed by Yokota et al. (2018).

Additionally, exposure to oxidative stress may result in increased expression of the AT1 receptor for angiotensin, and consequently, increased activity of NHE_3_ (Johns et al., 2011). In the present study, fructose intake reduced the FE of sodium, an effect that is possibly related to renal RAS activation, as explained above. Moreover, the FE of sodium in the control groups was higher in female rats than in male rats, confirming sex differences. In rodents, there are morphofunctional differences in the kidneys with respect to sex. The PCT is longer and contains more sodium transporters in males than in females, providing more significant fractional sodium reabsorption (Veiras et al., 2017). The greater sodium reabsorption in males may also be related to the stimulatory effect of androgens on NHE_3_ of PCT (Quigley, 2008). In contrast, females have a greater abundance of the sodium-chloride cotransporters (NCC) and epithelial sodium channels in distal nephrons, providing more significant saline excretion in females (Veiras et al., 2020). Studies with Sprague-Dawley rats have shown that sodium excretion in males is significantly lower than that in females for the same renal perfusion pressure (Khraibi et al., 2001); similar results were observed in SHR (Khraibi et al., 2001; Kalucki et al., 2020).

Regarding potassium excretion, we observed that females in the control group had higher potassium excretion than the control males; and fructose intake significantly reduced potassium excretion in both sex. Given that an increased urinary flow induces urinary potassium loss, it is possible that there was greater potassium excretion at the beginning of the experimental period; however, physiological mechanisms may have been activated throughout the treatment period to reduce potassium loss. The activation of NCC in the distal tubule, resulting in a lower sodium load to the collecting duct and, thus, reducing sodium reabsorption/potassium secretion in this nephron segment is a possible mechanism (McDonough and Youn, 2017). However, further experiments are needed to confirm the participation of this mechanism in the reduction of potassium excretion in this experimental model.

The renal excretion of other essential electrolytes transported in the renal tubules, such as calcium and magnesium, was also evaluated. The FE of calcium in females groups was higher than that in males. Most of the filtered calcium is reabsorbed in the PCT that is relatively shorter in females (Veiras et al., 2017) which may in greater excretion. This difference can also be attributed to the effects of sex hormones on ion-transport (Khalil et al., 2018). The role of androgens in the renal control of calcium homeostasis is controversial. Androgens were suggested to increase urinary calcium excretion, in view that decreased calcium excretion was observed 2 weeks after orchidectomy (Hsu et al., 2010). In contrast, other studies demonstrated that orchidectomy of male rats induced hypercalciuria at two (Lin et al., 2016) and 8 weeks (Gaumet-Meunier et al., 2000) after surgery. However, these results should be interpreted with caution, as serum calcium levels and calciuria are also influenced by other physiological mechanisms involving intestinal and bone exchange (Khalil et al., 2018).

Estrogen has been discussed to either directly affect the renal calcium reabsorption or indirectly influence it by altering calcium metabolism by other mechanisms. However, there is evidence that estrogen influences calcium metabolism *via* the upregulation of TRPV5 in the distal nephron (Van Abel et al., 2002). Thus, the effects of sex hormones on renal calcium transport are complex, and further studies are required to clarify this issue (Khalil et al., 2018).

Urinary magnesium excretion was higher in female fructose-treated rats. Magnesium is mainly reabsorbed in the thick segment of the loop of Henle depending on the lumen’s positive electrical potential established by the secretion of potassium through the ROMK channel, which in turn depends on potassium transport by the NKCC (de Baaij et al., 2012). In FF rats, the higher fluid intake may have decreased the release of antidiuretic hormone (ADH) that also increases NKCC transporter activity on Henle’s loop (Ecelbarger et al., 2001); and in this way, may have indirectly reduced magnesium reabsorption. Furthermore, it seems that ADH also exerts a stimulatory effect on magnesium reabsorption in the distal nephron (Dai et al., 2001). Estrogen has as well been shown to increase magnesium reabsorption in the distal nephron by activating the transient receptor potential cation channel 6 (TRPM6) (Groenestege et al., 2006). However, there are no reports on the possible influence of androgen hormones on tubular magnesium reabsorption.

The high-fructose diet altered the blood urea concentration. In the fructose-treated groups, a larger fluid intake inhibited the release of ADH in the pituitary glands. In addition to promoting water reabsorption in the distal nephron, ADH increases urea reabsorption in the medullary collecting duct (Sautin et al., 2007); therefore, the reduction of plasma ADH favors the excretion of urea, reducing the concentration of urea in the blood of the rats in the fructose groups. Moreover, fructose also modulates hepatic urea synthesis, contributing to reduced blood urea levels in the treated groups (Shortliffe et al., 2015).

Regarding the renal morphological assessment, higher glomerular area values were found in the male groups. However, the literature on sexual dimorphism in this parameter is controversial. There are reports that men have a larger glomerular area (McLachlan et al., 1977; Nyengaard and Bendtsen, 1992; Kalucki et al., 2020), while other do not show differences between sexes (Baylis, 1994). In contrast, experimental studies indicate a greater glomerular area in males (Gafter et al., 1990; Sakemi and Baba, 1993; Alberti et al., 2016). Baylis (1994) evaluated glomerular volume in male and female rats of different ages and verified that females have a smaller glomerular volume at all ages. They also showed that the development of kidney disease is associated with the presence of androgens, but not necessarily with glomerular enlargement (Baylis, 1994). This association may be linked to alterations in the renin-angiotensin-aldosterone system, as plasma renin levels and activity are higher in male than in female rodents (Chen et al., 1992). Furthermore, the renal vasculature of male rats appears to be more sensitive to ANG II (Wangensteen et al., 2004). Factors that reduce NO production may further accentuate the vasoconstrictor effect of ANG II, thus accelerating the progression of kidney disease (Wangensteen et al., 2004).

Analysis of renal tissue revealed an increase in macrophage infiltration in the high-fructose group and the expression of the oxidative stress marker 8-OHdG, as well as reduced expression of the eNOS enzyme. Ingested fructose is absorbed by enterocytes and reaches the bloodstream. In hepatocytes, the ketoexokinase enzyme catalyzes fructose phosphorylation; this molecule undergoes the action of several enzymes, giving rise to uric acid (Choi et al., 2010). In kidney tissues, fructose triggers pro-inflammatory pathways that induce the production of monocyte chemoattractant protein-1 (MCP-1), the latter of which can also be increased by uric acid and ROS. This ROS production may originate from NADPH oxidase and/or xanthine oxidoreductase (Cirillo et al., 2009). Uric acid can cause eNOS uncoupling and reduce NO availability (Nakagawa and Kang, 2021). Our results confirmed that high fructose levels promoted macrophage migration to the kidneys, increased ROS production (estimated by an indirect measurement), and decreased eNOS enzyme expression. These alterations, possibly associated with uric acid production, may also be related to the upregulation of RAS components. Further experiments are required to confirm this hypothesis.

Furthermore, the expression of eNOS in the control groups was higher in female rats than in male rats, confirming sex differences. This result was expected because estradiol increases the activity of eNOS, and consequently the production of NO (Wyckoff et al., 2001). Several studies have indicated that the cardiovascular and renal benefits of female sex (before menopause) are mainly related to NO production. Under physiological conditions, the interaction between the NO and RAA systems results in the downregulation of Ang II type 1 receptor expression and the associated antagonism of Ang II generation (Yan et al., 2003; McGuire et al., 2007; Sharma et al., 2012), which may result in the protection of female kidney function.

## Conclusion

The introduction of fructose overload, starting from weaning, had important repercussions on the kidneys of rats of both sexes. Male rats that received this sugar had a significant reduction in the GFR. In females, the alterations were more tenuous; however, notable changes were observed in the electrolyte excretion. Both male and female rats showed increased macrophage infiltration and reduced eNOS expression, confirming the consequences of fructose metabolism in the kidneys.

## Data Availability

The datasets presented in this study can be found in online repositories. The names of the repository/repositories and accession number(s) can be found below: https://repositorio.unifesp.br.
